# Hardship financing of out-of-pocket payments in the context of free healthcare in Zambia

**DOI:** 10.1371/journal.pone.0214750

**Published:** 2019-04-10

**Authors:** Oliver Kaonga, Charles Banda, Felix Masiye

**Affiliations:** Department of Economics, School of Humanities and Social Sciences, University of Zambia, Lusaka, Zambia; Paediatric Centre of Excellence, ZAMBIA

## Abstract

Despite the removal of user fees on public primary healthcare in Zambia, prior studies suggest that out-of-pocket payments are still significant. However, we have little understanding of the extent to which out-of-pocket payments lead patients to hardship methods of financing out-of-pocket costs. This study analyses the prevalence and determinants of hardship financing arising from out-of-pocket payments in healthcare, using data from a nationally-representative household health expenditure survey conducted in 2014. We employ a sequential logistic regression model to examine the factors associated with the risk of hardship financing conditional on reporting an illness and an out-of-pocket expenditure. The results show that up to 11% of households who reported an illness had borrowed money, or sold items or asked a friend for help, or displaced other household consumption in order to pay for health care. The risk of hardship financing was higher among the poorest households, female headed-households and households who reside further from health facilities. Improvements in physical access and quality of public health services have the potential to reduce the incidence of hardship financing especially among the poorest.

## Introduction

The general failure of cost sharing-based policies in healthcare that led to worsening conditions in terms of access and financial protection in health systems around Sub-Saharan African led to new health financing policy regimes. Some countries have experimented with a number of policies such as community-based health financing schemes, user fee abolition, prepayment schemes, social health insurance, or a mix of these [1, 2]. These policies were intended to guarantee greater healthcare access and financial protection, especially to the poorest who were worst hit by user charges. Arguably, the boldest of such policies has been the complete removal of user fees in the public sector, starting with primary healthcare which is seen to benefit the poorest more [3, 4]. Despite these policy efforts, out-of-pocket payments (OOPs) associated with a visit to a health provider remain high, and in some cases prohibitive, in Sub-Saharan Africa [5, 6]. African health systems rely heavily on OOPs as a source of financing for healthcare. In Zambia, healthcare seeking is associated with significant OOPs at the point of use in both public and private facilities.

In this paper, we focus not merely on the amount spent but also on identifying the difficulties or hardships represented by particular means by which money is mobilized to pay for out-of-pocket healthcare costs. Studies have identified ‘hardship financing’ as methods of coping with healthcare OOPs which cause adverse economic and health consequences at the household level [7–9]. In very low-income settings where incomes are seasonal and not fully monetary, the amount spent may be a misleading measure of financial hardship or affordability [5, 8]. However, empirical evidence on how households raise money to cope with burdensome out-of-pocket healthcare costs in free healthcare settings in developing countries is limited.

Our study relates, more generally, to recent literature evaluating the impacts of user fee removal and partial subsidies in healthcare on providing relief from high OOPs to all population groups. Broadly, these studies report that out-of-pocket costs have remained very high in many health systems contexts across sub-Saharan Africa [3, 4, 10, 11]. For example, following the removal of user fees in the Ugandan public health system, quality deteriorated so much so that patients shifted to private sector where they encountered even higher OOPs. Consequently, the poor continued to experience high levels of catastrophic health expenditure (CHE) or constrained access to necessary medical care [10].

In 2012, the government of Zambia extended the policy of free public primary healthcare, which was initially implemented in rural primary health facilities in 2006, to all areas in the country. The goal of this policy was to eliminate financial barriers to access and increase utilization of primary healthcare [12]. The experience in Zambia was slightly different from Uganda’s case. Removal of user fees provided financial relief through free healthcare to users [13]. Lepine et al [14] also demonstrated that financial relief from free healthcare in Zambia went disproportionately to well-off households, with no impact on access to care for the poorest. Obare et al [15] showed that the removal of user fees did not benefit the poorest, and neither did it deliver a sustained and significant impact on financial protection and access to maternal health services. Other cross-country studies have also reported that financial protection from OOPs is limited even in systems where no patient charges exist [4, 7].

This study seeks to achieve two main objectives: (i) to examine the extent to which patients resort to hardship financing means of meeting OOPs, and (ii) identify factors associated with likelihood of experiencing hardship financing of OOPs. In line with literature, our empirical indicators of hardship financing consider whether healthcare costs are so high as to cause patients to adopt any of the following strategies: (a) borrow money or sell assets in order to meet out-of-pocket payments, (b) healthcare OOPs substitute expenditure on other basic essential consumption, (c) ask a friend to meet own OOPs, and (d) avoid OOPs costs by foregoing healthcare utilization. [6, 16–19]. The study used data from a 2014 nationally-representative household health expenditure and utilization survey to determine the extent of financing hardships associated with outpatient health utilization, and associated determinants.

### The zambian health system context

In this section, we provide a brief profile of the Zambian healthcare system. The ownership structure is such that, 79% of health facilities are owned by government, 2.3% by faith-based organizations (mission facilities) and 19% are private-for-profit [20]. The majority of the population (86%) utilise public health facilities. The private sector and mission facilities account for about 3% and 6% of healthcare visits respectively. Traditional healthcare accounts for the remaining 5% [21]. In terms of health expenditure, the country’s per capita total health expenditure is estimated at about US$60.00. The Government and Donors account for almost an equal share (41% and 42% respectively) of the current health expenditure. Out-of-pocket health expenditure contributes 12.8% to total health expenditure. Private medical schemes and insurance account for about 5% [22].

Zambia’s public health system is structured in a four-tier pyramidal referral set-up, with primary healthcare at the bottom. Primary healthcare consists of a network of health posts, health centres and district hospitals managed under the District Health Office (DHO). Above the primary healthcare level are second level hospitals. Second level hospitals were designed to serve as the highest referral hospitals at provincial level. The next level of care are the third level hospitals which provide tertiary level healthcare and in some cases medical training and research. Finally, at the apex of the health system are specialised hospitals.

## Methods

### The sample and data

The 2014 Zambian Household Health Expenditure and Utilisation Survey (ZHHEUS) is a cross-sectional survey conducted by the Central Statistical Office and the University of Zambia with support from the Ministry of Health. The data were collected using a two-stage stratified cluster sample design. In the first stage, standard enumeration areas were selected within each stratum using the probability-proportional-to-estimated-size procedure. In the second stage, a fixed proportion of 20 households were selected from each enumeration area using a systematic random sampling procedure. The data was collected from 12,000 households sampled from across all the ten provinces in the country. For all selected households, data was collected on each household member giving a total of about 60,000 individual observations. The survey response rate was 99.4%.

Individuals were asked if they had experienced an illness or injury in the 4 weeks preceding the survey, or if they had been admitted to a health facility in the 6 months preceding the survey. The survey included questions on health status (self-rated health status and self-reported illness experience); healthcare utilisation (visits, admission, and type of providers sought), and health expenditure (amount spent during visit or admission, item on which amount was spent, and source of funds). Out-of-Pocket health expenditure included expenditure at the facility (for drugs, consultation, medical examinations, etc.), medical expenditure outside the facility (e.g. for drugs purchased at a private Drug Store rather than the facility visited), non-medical costs associated with seeking healthcare (e.g. travel costs, food, lodging, etc.). This study focused on analyzing hardship financing related to outpatient visits rather than admissions. The data on out-of-pocket payments for admissions was very noisy. For example, of those who were admitted, 92% reported zero expenditure. We strongly believe that this data suffered from recall bias.

The survey included several socioeconomic and demographic variables. Household income was measured indirectly using consumption expenditure in the one month prior to the survey, as is common in similar surveys in low income countries. For education of the head of the household, the survey asked the highest level of schooling completed, while employment type was categorized as either employed or unemployed. Demographic variables on individuals in each household, including age, sex, marital status, residence, employment status, and so on, were also captured. A number of these individual and household characteristics were included as covariates as shown in Table 1.

**Table 1:** Descriptive statistics of the sample.

Variable Name	Number(n)	%
Region of residence is rural	6,763	60.21
Sex of Head of Household is male	8,781	75.40
Level of Education of Head of Household		
No formal education	2481	21.39
Primary	5550	47.73
Secondary	3063	26.32
Tertiary	536	4.56
Employment Status of Head of Household		
Employed	9,826	78.77
Not employed	2496	21.23
	**Mean**	**SD**
Per capita monthly Household expenditure in 2014 Kwacha	214.7	498.61
Age of sample participants in years	21.7	17.89

### Measures of hardship financing for OOPs

In line with the literature, we define a measure of ‘hardship’ financing as methods of raising money for out-of-pocket payments that either signal or induce financial difficulty at the household level. A household is considered to have experienced hardship financing if they reported financing healthcare OOPs using any of the following four mutually-exclusive options: (i) sold assets or borrowed money, (ii) asked a relative or friend to pay, (iii) experienced catastrophic level of OOPs, and (iv) did not seek care in avoidance of unaffordable OOPs [7, 8, 23, 24].

The empirical literature provides justification for the inclusion of the indicators of hardship financing. For example, selling assets or borrowing money are considered hardship means of coping with out-of-pocket healthcare costs because of the economic hardships that both strategies impose on households [9]. While reliance on the generosity of friends or relatives might reflect presence of some degree of social capital, it is most likely that the individuals rather faced financial hardships. The phenomenon of households asking relatives or friends to defray health costs is commonly associated with financial distress due to illness [25–27]. Further, as identified by Xu et al [28], CHE presents a form of hardship financing because CHE often implies that households have to forgo or compromise other basic household consumption such as food in order to pay for healthcare. Another study has used CHE as an indicator of hardship financing [29]. And, a household forced into CHE by healthcare OOPs is likely to get into debt or poverty. CHE is calculated as OOPs per visit that exceeds 10% of total household expenditure [2, 30]. Finally, forgoing treatment on account of cost is a strategy that indicates that a household faces a real prospect of getting into financial distress due to OOPs.

### Statistical model of determinants of hardship financing

Our statistical analysis is divided into two parts. We first run a two-stage sequential response model to demonstrate the association between the likelihood of experiencing hardship financing and a host of individual demographic and socio economic characteristics conditional on an individual reporting an illness and incurring some positive out-of-pocket payment. A sequential response model is a statistical method used to analyse sequential decisions or events [31]. The model assumes that individuals make choices, but often these choices are not made simultaneously but rather sequentially. The sequential response model is essentially a sequence of binary-outcome models that could be either logit or probit models. Exceptions do exist in which a stage in the sequence is another probability model such as a multinomial-response model [32]. The sequential model estimated in this paper combines binary and multinomial logistic models.

In the first stage of the sequential response model, an individual who has visited a health provider is faced with two alternatives, either they pay or do not pay. This means the group of individuals who decide to seek care belong to one of the two categories; paying or not paying. Given only two options we estimate the binary logit model specified as

$$(\mathrm{Stage}\;1)\;P(y\in A_{h})=\frac{{\exp(x}^{'}\beta_{h})}{1+{\exp(x}^{'}\beta_{h})}\;\mathrm{for}\;h=1,2$$

where; h = 1 if the individual did not pay for healthcare and h = 2 if the individual paid to access health services.

In the second stage, we estimate a multinomial logistic regression. Individuals who visited a health provider and paid to access health services are faced with one or more of the scenarios specified in the model below. For estimation purposes, we use the category of individuals who paid for health services using own cash without incurring Catastrophic Health expenditure as the reference category.

$$(\mathrm{Stage}\;2)\;P(y=j/A_{h})=\frac{\exp(x^{'}\beta_{j})}{\sum k\in A_{h}\exp(x^{'}\beta_{k})}\;\mathrm{where}\;j=0,1,2,3,4$$

j = 0 if the individual paid for healthcare using own cash without incurring Catastrophic Health expenditure

j = 1 if the individual borrowed money or sold assets to pay for healthcare

j = 2 if the individual requested someone else to pay for them

j = 3 if the individual paid above the threshold considered above Catastrophic healthcare expenditure (CHE)

Finally, the second part of our statistical analysis estimates a multinomial logit model in order to analyse the factors that are associated with the decision to seek care or not to seek care. All analyses were conducted using STATA/SE 13.0.

### Ethical considerations

Since the survey did not involve collection of human samples, ethical exemption for this study was granted under the provisions of the Census and Statistics Act Number 127 of the laws of Zambia. No identifying information of individuals or health institutions were collected in the survey. In observance of the ethical requirements, only participants aged at least 15 years were interviewed after giving written (signature or thumb print) informed consent.

## Results

### Descriptive statistics of the sample

Table 1 summarises the characteristics of sampled individuals. The majority of households are headed by the males who are the primary decision makers of the household. Consistent with other national surveys, about 60% of the population reside in rural areas [33, 34]. Education levels are generally low with more than half having either no formal schooling or only primary education. About 79% of heads of households were in employment (salaried or self-employed) while the rest were classified as unemployed (e.g. student, home-marker, aged, etc.). The average nominal per capita monthly household expenditure is Kwacha 214.7 (equivalent of US$21.47).

### Health service utilization and hardship financing of OOPs

In Table 2 we present results on illness experience, healthcare seeking options, out-of-pocket expenditure and measures of hardship financing. About one fifth of the sample reported an illness or injury in the four weeks preceding the survey. Of the persons who reported illness or injury, 62% visited a health facility while 29% self-medicated. Furthermore, 9% could not seek care due to cost, illness not being serious, religious reasons, etc. More than half of the respondents who reported an illness four weeks prior to the survey suffered from either Malaria or Fever. Other prominent illnesses reported include Diarrhea (10%), Respiratory infections (4%) and headache (5%).

**Table 2:** Health Service utilization and hardship financing of OOPs.

Variable name	Number (n)	%
Fell sick in past 4 weeks	13,150	22.13
Care options following illness		
Sought care	8,146	61.85
Self-medicated	3,814	29.10
Did nothing	1,191	9.05
Type of illness reported		
Malaria/fever	6,962	52.94
Respiratory infections	512	3.89
Headache	676	5.14
Diarrhea	1,324	10.06
Other illnesses	3,676	27.95
Type of provider visited		
Public Hospital	1,020	12.52
Public Health Centre	4,526	55.56
Public Health post	1,502	18.43
Mission Facility	467	5.74
Private Facility	251	3.08
Other Facility types	380	4.66
Paid nothing during visit	5,496	67.55
Indicators of Hardship financing of OOPs (with transport costs)		
Borrowed or sold assets	70	0.50
Asked someone else to pay	318	2.40
Paid above CHE threshold	1,113	8.46
Composite measure of hardship financing	1,501	11.40
Indicators of Hardship financing of OOPs (without transport costs)		
Borrowed or sold assets	33	0.25
Asked someone else to pay	185	1.41
Paid above CHE threshold	446	3.39
Composite measure of hardship financing	664	5.05
Could not seek care due to cost	988	7.50
	**Mean**	**SD**
Amount of OOPs per visit in Kwacha	14.90	165.50
Distance to nearest health facility in Kilometres	5.20	13.01

The majority of the patients visited health centers and health posts. Only 3% visited private facilities. In Table 3, the Pearson's correlation test shows that household income (proxied by household consumption expenditure) is positively associated with choosing a private health facility, which suggests that the poor are more likely to choose public facilities. Similarly, visiting a private facility is positively correlated with the level of education. An association is also found between choosing a private facility and urban residence, whereby the urban residents are more likely to visit private facilities. Private health facilities are almost exclusively located in urban areas. One patient in every five incurred health expenditure during visits to a healthcare provider. The average expenditure per visit is K14.90 (US$1.49), with a standard deviation of 166. The large standard deviation reflects the wide variations in the amounts of OOPs. Some of the larger amounts of OOPs were reported in public hospitals and private facilities.

**Table 3:** Association between health provider choice and Household characteristics.

Variable	*Pearso*n’s Correlation coefficient
Health Facility type(1 = Public, 2 = Private)	
Sex of head of household (Male = 1, Female = 2)	-0.01420
Age of head of household	0.0482**
Education level of household head	0.1854**
Region of residence (Rural = 1, Urban = 2)	0.1907**
Employment (Unemployed = 1, Employed = 2)	0.1910**
Household expenditure	0.3146**

**P < .05 (significant at 5%)

Overall, the results show that 11.4% of households who incurred health expenditure faced one form of hardship financing or another. When transportation costs are excluded, the incidence of hardship financing reduces to about 5%, showing that transportation costs are a major part of healthcare seeking costs. The major source of hardship financing was CHE (8.5%). About 3% either asked someone else to pay or borrowed or sold assets to meet their out-of-pocket expenditure. Further, it is also noteworthy that 7.5% of those who reported an illness did not seek care due to cost, which likely indicates avoidance of financial hardships.

Table 4 presents the results of the sequential logit model estimation of the association between individual characteristics and the likelihood of facing any of the forms of hardship financing. The first part of the model shows the characteristics that affect an individual’s likelihood of making a positive out-of-pocket expenditure. Household consumption expenditure is associated with significantly increased odds (or likelihood) of incurring a positive out-of-pocket health expenditure (OR = 1.37). Residing in the urban region is associated with increased odds of paying OOPs compared to rural regions. Paying for health services is more prevalent in urban areas because urban areas tend to have better quality health services and more facilities such as pharmacies. The result could reflect the fact that monetised transactions are limited in rural areas. Additionally, distance to a health facility is found to be positively associated with higher odds of incurring OOPs. The results further show that paying for health services is more likely among those who visited a public health centre and public health posts, compared to those who visited a public hospital, whereas the odds increase with visiting a private health facility. The odds of making payments for outpatient services at primary facilities are lower because of the policy of free healthcare.

**Table 4:** Predictors of hardship financing of OOPs—Sequential response model results.

	Paying vs not paying		Sold assets/borrowed vs regular income non-CHE		Requested Someone else to pay vs regular income non-CHE		CHE vs regular income non-CHE	
Variable name	OR	SE	OR	SE	OR	SE	OR	SE
Age	1.01***	0.00	1.00	0.01	0.99***	0.00	1.00	0.00
Sex (1 = Female 0 = Male)	0.96	0.05	1.15	0.38	1.44***	0.21	1.27**	0.12
Region of residence(0 = rural, 1 = urban)	1.18**	0.08	0.87	0.36	1.25	0.22	1.14	0.14
Distance to health facility	1.01***	0.00	1.03***	0.01	1.02***	0.01	1.02***	0.00
Log of per capita Household expenditure	1.37***	0.03	0.67**	0.11	0.54***	0.04	0.39***	0.02
In employment (1 = employed, 0 = Not employed)	0.94	0.07	0.93	0.48	0.90	0.17	1.10	0.15
No formal education (Reference category)								
Primary education	1.10	0.10	1.11	0.63	1.11	0.33	0.78	0.15
Secondary education	0.98	0.10	0.44	0.29	1.29	0.39	0.83	0.16
Tertiary education	1.06	0.14	0.20	0.20	1.40	0.52	1.02	0.25
Public hospital (Reference category)								
Public health centre	0.69***	0.05	0.45	0.2*	0.61***	0.11	0.41***	0.06
Public health post	0.43***	0.04	0.77	0.42	0.23***	0.08	0.44***	0.08
Mission health facility	0.57***	0.07	0.43	0.35	0.59	0.21	0.58**	0.14
Private health facility	1.68***	0.27	3.69	2.51*	2.27***	0.71	2.51***	0.57
Malaria/fever (Reference category)								
Respiratory illnesses	1.21	0.16	0.92	0.96	1.46	0.51	1.29	0.32
Diarrhea	1.02	0.13	0.79	0.82	1.22	0.43	1.39	0.34
Headache	1.01	0.10	0.52	0.54	0.87	0.29	1.22	0.24
Other illness types	1.32***	0.08	2.44**	0.87	1.86	0.31***	2.11***	0.23
Constant	0.12***	0.02	0.35	0.39	2.56	1.29*	61.76***	21.70

Number of obs = 7,585; Prob > chi2 = 0.000; Log likelihood = -7071.74; LR chi2 (48) = 1453.72

***P < .01 (significant at 1%)

**P < .05 (significant at 5%)

*P < .1 (significant at 10%).

In Table 4, we also present results which show the factors that are associated with the likelihood of households to resort to one form of hardship financing or another in coping with out-of-pocket expenses, conditional on having incurred an out-of-pocket payment. Higher household income (as measured by consumption expenditure) is associated with a lower likelihood of incurring hardship financing. For each unit increase in log-transformed per capita expenditure (which is equivalent to K2.72 in natural kwacha terms), the odds of borrowing/selling assets or asking someone else to pay or making catastrophic OOPs decrease by 33%, 46% and 59%, respectively. This means that households with less resources are more prone to borrowing or selling assets to meet healthcare payments. Also, in such households, facing unaffordable OOPs increases the odds that healthcare OOPs will displace spending on other important basic households needs. Further, the odds of borrowing or selling assets or relying on someone else to help meet OOPs or making catastrophic OOPs are predicted to significantly increase with the distance to the health facility. For each additional kilometer travelled to the facility, the odds of experiencing hardship financing increases by 2–3%, across the three types of hardship financing.

In addition, the results show that after household expenditure and other variables are controlled for, female-headed households are significantly more likely to depend on asking someone else to meet unaffordable healthcare payments (OR = 1.44) and making payments which are catastrophically high (OR = 1.27). Visiting a private health facility was associated with a higher likelihood of facing financing hardships in the form of a patient requesting someone else to pay for them. The log odds of requesting someone else to pay are significantly greater (OR = 2.3) for those who visited private health facilities, compared with those who visited public hospitals. Similarly, visiting primary healthcare facilities (health centres and health posts) was associated with a reduced likelihood of requesting someone else to pay. Households who visited public health centres, health posts or mission hospitals had a reduced likelihood of facing CHE while those who visited private facilities had a significantly higher likelihood of experiencing CHE, compared to those who visited public hospitals. Other factors such as the region of residence, level of education and the type illness are found to be non-significant predictors of experiencing hardship financing.

Table 5 shows the results of the multinomial logistic regression of factors associated with an individual’s inability to seek care on account of cost. The results show that living in an urban area increases the likelihood of failing to seek care on account of cost. Higher household expenditure is associated with reduced likelihood of failing to seek care on account of cost. Higher level of education is associated with lower likelihood of failing to seek care on account of cost.

**Table 5:** Foregoing seeking care due to Cost-Multinomial Logistic regression results.

*Sought care* : *Base category*	Did not seek care due to cost		Did not seek care due to other reasons including self-medication	
Variable name	RRR	SE		RRR	SE
Age	0.99	0.01	1.00	0.00
Sex (male = 0, female = 1)	0.81	0.29	0.72***	0.06
Region of residence(0 = rural, 1 = urban)	2.55**	1.00	1.06	0.10
Log of per capita household expenditure	0.77	0.10	0.97	0.03
Employed(1 = paid emp, 0 = otherwise)	0.96	0.35	1.10	0.10
No formal education (Reference category)				
Primary	0.23***	0.09	0.73***	0.09
Secondary	0.11***	0.06	0.65***	0.09
Malaria/fever (Reference category)				
Respiratory	0.82	0.63	0.93	0.18
Diarrhea	0.61	0.64	1.93***	0.34
Headache	0.71	0.45	1.78***	0.21
Other illnesses	0.90	0.32	0.99	0.08
Constant	0.13**	0.13	1.32	0.32

Abbreviations: RRR = Relative Risk Ratio; SE = Standard error

Number of obs = 12486; Prob > chi2 = 0.0000; Log likelihood = -8889.405; LR chi2 (24) = 345.16

***P < .01 (significant at 1%)

**P < .05 (significant at 5%)

*P < .1 (significant at 10%).

In terms of factors associated with the likelihood of not seeking care due to non-cost factors, it is shown that income is not significant. Being from a female-headed household is significantly associated with a lower probability of failing to seek care due to non-cost reasons such as religious or cultural beliefs, illness not being serious, self-medication, etc. Patients from households headed by individuals without any formal education was associated with a higher likelihood of failing to seek care due to reasons other than cost. Patients of diarrhea and headache were less likely to seek care due to non-cost reasons compared to malaria patients.

## Discussion

This study has examined the extent and nature of financial hardships experienced by patients due to unaffordable out-of-pocket healthcare payments in Zambia. Overall, we estimate that about 11% of patients who reported a visit experienced financial distress associated with OOPs, which by any account indicates a significant prevalence of unaffordable burden of OOPs. Hardship financing was exemplified by borrowing money, asking someone else to pay for them, or selling personal or household items, and CHE, which entails that OOPs displaces basic household consumption. This level of hardship financing is much lower than what was reported in a study by Tahsina et al [35], mainly because of the removal of user fees, though similar to a study in Bangladesh.

Furthermore, findings from the sequential logit model shades light on socioeconomic factors which are associated with the likelihood of experiencing one form of financing hardship or another: having a low household income, visiting a private facility or hospital, being under a female-headed household, and living further from a health facility. These findings have been corroborated and established in a number of past studies [7, 36]. For example, studies have shown that households with severely limited incomes or resources are associated with increased likelihood of facing financial distress in meeting healthcare payments. What we also find interesting is that female-headed households are more likely to experience financial hardships in form of CHE (because they have very little income to start with), or relying on someone else to meet their healthcare costs. They often do not have capacity to borrow money or have assets to sell. Vulnerability of female-headed households to financing hardships is rooted in gender-based structural characteristics of the Zambian society. In our data, two thirds (66%) of heads in female-headed households are either widows or divorcees. In patriarchal societies such as Zambia, a woman loses access to productive household assets, such as land, housing, animals, cars, etc., when her husband dies or when she gets divorced [37]. A study in Nigeria reported that female headed-households reported higher cost burdens and affordability problems [38].

A number of factors explain, at least partially, the level of hardship financing of OOPs in Zambia. First, hardship financing is likely occasioned by gaps in the facility healthcare quality especially in terms of shortage of drugs, inadequate medical examination facilities and overcrowding. Studies have shown that good quality of facilities is of central importance for realizing the potential financial relief of free public health services [11, 39, 40]. Further, as Nabyonga et al [10] observed, poor patients can also be forced to go to more expensive private facilities or hospitals in fear of poor quality of care in primary facilities.

Second, it is clear from the analysis that costs of travel to health facilities, and related expenses, which constitute a significant portion of total healthcare OOPs do cause financing hardships. These hardships are generally higher in rural areas where distances are longer. Vulnerability to distress associated with unaffordable OOPs has been shown to increase with distance from a facility [36]. Third, patients may face hardships when they choose to go to private facilities which charge much higher user fees. Although in interpreting this association, we were unable to control for the selection of poorer patients into private facilities for outpatient care. It seems reasonable to assume that patients of limited financial capacities would decide to go to public facilities. Thus, the likelihood of hardship financing is generally associated with use of private facilities. However, even poor patients in urban areas do visit private facilities because of the perception that public healthcare is of poor quality [41, 42].

Another possible source of high OOPs is that health staff at the primary facilities which are designated to provide care could introduce quasi-formal user charges as a response to insufficient funding by the government for medical and non-medical supplies. Future studies should explore the issue of informal or quasi-formal healthcare payments. A study by Damme et al [43] in Cambodia reported similar findings. Unfortunately, the survey did not ask respondents if they were aware that primary healthcare is free in the public sector.

From a policy perspective, the evidence of vulnerability to hardship forms of coping with unaffordable OOPs highlights the need for policy attention to the ongoing problem of hardship financing in healthcare. Although in this study we have not investigated the health and welfare implications of hardship financing on households, studies have shown how various forms of hardship financing have caused impoverishment [8, 44]. Similarly, financial hardships have also been reported to cause patients to take inadequate treatment [45–47]. In order to realise the policy goal of universal protection against financial catastrophe of OOPs, there is a need for not only increased funding for public health services but also more equitable allocation of those resources. A study by Lepine et al [14] demonstrated that healthcare benefits and financial relief from free healthcare in Zambia accrue disproportionately to well-off households. When healthcare resources become scarce, the poorest will bear a disproportionate burden of financial distress of OOPs [48, 49].

Finally, this study has a number of limitations. First, household surveys are subject to recall bias in reporting out-pocket expenditure which could affect our calculation of CHE-based hardship financing. Second, self-reported illness is also susceptible to recall bias. Third, we are unable to establish the quality of care received by patients. For example, if patients who reported zero expenditure went without adequate treatment, it means we underestimate the true burden of financial hardship. The fourth and final limitation is that the survey did not ask about the source of borrowing (i.e. whether these were commercial or interest free loans) or the amount borrowed. Local money lenders charge so high interest that the amount to be repaid is much higher than the amount borrowed. Such information could be useful to analyse more fully the level of financing hardship imposed by health payments. These limitations notwithstanding, the findings from this study are consistent with similar studies on financial hardships associated with healthcare visits.

## Conclusion

Despite the removal of user fees on primary healthcare in the public sector, this study estimates that up to 11% of Zambian households who experienced an illness still experienced one form of hardship financing or another when coping with out-of-pocket healthcare payments. Furthermore, the disproportionate share of the burden of hardship financing falls on households with very low incomes, female-headed households, and individuals with less education. These findings demonstrate the ongoing policy challenge of reducing financial catastrophe associated with OOPs in Zambia. Finally, the results suggest that improving physical access and quality of care (particularly availability of drugs) in public facilities would likely reduce the incidence of hardship financing especially among the poorest.

## References

[1] Etienne C, Asamoa-Baah A, Evans DB. Health systems financing: the path to universal coverage: World Health Organization; 2010. 10.2471/BLT.10.078741 PMC2878164 20539847

[2] World Health Organization. State of health financing in the African Region. 2013.

[3] James CD, Hanson K, McPake B, Balabanova D, Gwatkin D, Hopwood I, et al To retain or remove user fees? Applied health economics and health policy. 2006;5(3):137–53. 10.2165/00148365-200605030-00001 17132029

[4] McPake B, Brikci N, Cometto G, Schmidt A, Araujo E. Removing user fees: learning from international experience to support the process. Health policy and planning. 2011;26(suppl_2):ii104–ii17. 10.1093/heapol/czr064 22027915

[5] World Bank. Health Equity and Financial Protection Data Sheet, Ethiopia. 2012.

[6] Lagarde M, Palmer N. The impact of user fees on access to health services in low‐and middle‐income countries. Cochrane Database of Systematic Reviews. 2011;(4). 10.1002/14651858.CD009094 21491414 PMC10025428

[7] Leive A, Xu K. Coping with out-of-pocket health payments: empirical evidence from 15 African countries. Bulletin of the World Health Organization. 2008;86:849–56C. 10.2471/BLT.07.049403 19030690 PMC2649544

[8] Kruk ME, Goldmann E, Galea S. Borrowing and selling to pay for health care in low-and middle-income countries. Health Affairs. 2009;28(4):1056–66. 10.1377/hlthaff.28.4.1056 19597204

[9] Binnendijk E, Koren R, Dror DM. Hardship financing of healthcare among rural poor in Orissa, India. BMC health services research. 2012;12(1):23 10.1186/1472-6963-12-23 22284934 PMC3317855

[10] Nabyonga Orem J, Mugisha F, Kirunga C, Macq J, Criel B. Abolition of user fees: the Uganda paradox. Health policy and planning. 2011;26(suppl_2):ii41–ii51. 10.1093/heapol/czr065 22027918

[11] Laokri S, Weil O, Drabo KM, Dembelé SM, Kafando B, Dujardin B. Removal of user fees no guarantee of universal health coverage: observations from Burkina Faso. Bulletin of the World Health Organization. 2013;91:277–82. 10.2471/BLT.12.110015 23599551 PMC3629451

[12] Masiye F, Chitah BM, McIntyre D. From targeted exemptions to user fee abolition in health care: experience from rural Zambia. Social science & medicine. 2010;71(4):743–50. 10.1016/j.socscimed.2010.04.029 20542363

[13] Masiye F, Kaonga O. Determinants of healthcare utilisation and out-of-pocket payments in the context of free public primary healthcare in Zambia. International journal of health policy and management. 2016;5(12):693 10.15171/ijhpm.2016.65 28005549 PMC5144876

[14] Lépine A, Lagarde M, Le Nestour A. How effective and fair is user fee removal? Evidence from Zambia using a pooled synthetic control. Health economics. 2018;27(3):493–508. 10.1002/hec.3589 29034537 PMC5900920

[15] Obare F, Abuya T, Matanda D, Bellows B. Assessing the community-level impact of a decade of user fee policy shifts on health facility deliveries in Kenya, 2003–2014. International journal for equity in health. 2018;17(1):65 10.1186/s12939-018-0774-4 29801485 PMC5970478

[16] Russell S. The economic burden of illness for households in developing countries: a review of studies focusing on malaria, tuberculosis, and human immunodeficiency virus/acquired immunodeficiency syndrome. The American journal of tropical medicine and hygiene. 2004;71(2_suppl):147–55. 10.4269/ajtmh.2004.71.147 15331831

[17] Saksena P, Hsu J, Evans DB. Financial risk protection and universal health coverage: evidence and measurement challenges. PLoS medicine. 2014;11(9):e1001701 10.1371/journal.pmed.1001701 25244520 PMC4171370

[18] Bonfrer I, Gustafsson-Wright E. Health shocks, coping strategies and foregone healthcare among agricultural households in Kenya. Global public health. 2017;12(11):1369–90. 10.1080/17441692.2015.1130847 26785165

[19] Gustafsson-Wright E, Janssens W, Van Der Gaag J. The inequitable impact of health shocks on the uninsured in Namibia. Health policy and planning. 2010;26(2):142–56. 10.1093/heapol/czq029 20668002

[20] Ministry of Health. The 2017 List of Health Facilities in Zambia. 2017.

[21] Ministry of Health. Zambia Household Health Expenditure and Utilisation Survey. 2015.

[22] Ministry of Health. National Health accounts. Ministry of Health 2016.

[23] Sauerborn R, Adams A, Hien M. Household strategies to cope with the economic costs of illness. Social science & medicine. 1996;43(3):291–301. 10.1016/0277-9536(95)00375-4 8844932

[24] Flores G, Krishnakumar J, O'donnell O, Van Doorslaer E. Coping with health‐care costs: implications for the measurement of catastrophic expenditures and poverty. Health economics. 2008;17(12):1393–412. 10.1002/hec.1338 18246595

[25] McIntyre D, Thiede M, Dahlgren G, Whitehead M. What are the economic consequences for households of illness and of paying for health care in low-and middle-income country contexts? Social science & medicine. 2006;62(4):858–65. 10.1016/j.socscimed.2005.07.001 16099574

[26] DiFazio R, Vessey J. Nonmedical out-of-pocket expenses: a hidden cost of hospitalization. Journal of Pediatric Nursing. 2011;26(1):78–84. 10.1016/j.pedn.2010.01.010 21256415

[27] Emanuel EJ, Fairclough DL, Slutsman J, Alpert H, Baldwin D, Emanuel LL. Assistance from family members, friends, paid care givers, and volunteers in the care of terminally ill patients. New England Journal of Medicine. 1999;341(13):956–63. 10.1056/NEJM199909233411306 10498492

[28] Xu K, Evans DB, Kawabata K, Zeramdini R, Klavus J, Murray CJ. Household catastrophic health expenditure: a multicountry analysis. The lancet. 2003;362(9378):111–7. 10.1016/S0140-6736(03)13861-5 12867110

[29] Wagstaff A, Flores G, Hsu J, Smitz M-F, Chepynoga K, Buisman LR, et al Progress on catastrophic health spending in 133 countries: a retrospective observational study. The Lancet Global Health. 2018;6(2):e169–e79. 10.1016/S2214-109X(17)30429-1 29248367

[30] Wagstaff A. Measuring financial protection in health: The World Bank; 2008.

[31] Powers D, Xie Y. Statistical methods for categorical data analysis: Emerald Group Publishing; 2008.

[32] Liao TF. Interpreting probability models: Logit, probit, and other generalized linear models: Sage; 1994.

[33] Office CS. Living Conditions Monitoring Survey Report, 2015. 2016.

[34] Central Statistical Office [Zambia] MoHZ, and ICF International. Zambia Demographic and Health Survey 2013–14. 2014.

[35] Tahsina T, Ali NB, Siddique MAB, Ahmed S, Rahman M, Islam S, et al Determinants of hardship financing in coping with out of pocket payment for care seeking of under five children in selected rural areas of Bangladesh. PloS one. 2018;13(5):e0196237 10.1371/journal.pone.0196237 29758022 PMC5951548

[36] Cleary S, Birch S, Chimbindi N, Silal S, McIntyre D. Investigating the affordability of key health services in South Africa. Social science & medicine. 2013;80:37–46. 10.1016/j.socscimed.2012.11.035 23415590

[37] Chapoto A, Jayne T, Mason NM. Widows’ land security in the era of HIV/AIDS: Panel survey evidence from Zambia. Economic development and cultural change. 2011;59(3):511–47. 10.1016/j.socscimed.2012.11.035 21744545

[38] Onah MN, Govender V. Out-of-pocket payments, health care access and utilisation in south-eastern Nigeria: a gender perspective. PLoS One. 2014;9(4):e93887 10.1371/journal.pone.0093887 24728103 PMC3984110

[39] Masiye F, Kaonga O, Kirigia JM. Does user fee removal policy provide financial protection from catastrophic health care payments? Evidence from Zambia. PloS one. 2016;11(1):e0146508 10.1371/journal.pone.0146508 26795620 PMC4721670

[40] Pariyo GW, Ekirapa-Kiracho E, Okui O, Rahman MH, Peterson S, Bishai DM, et al Changes in utilization of health services among poor and rural residents in Uganda: are reforms benefitting the poor? International Journal for Equity in Health. 2009;8(1):39 10.1186/1475-9276-8-39 19909514 PMC2781807

[41] Fotso JC, Mukiira C. Perceived quality of and access to care among poor urban women in Kenya and their utilization of delivery care: harnessing the potential of private clinics? Health policy and planning. 2011;27(6):505–15. 10.1093/heapol/czr074 22080515

[42] Kiguli J, Ekirapa-Kiracho E, Okui O, Mutebi A, MacGregor H, Pariyo GW. Increasing access to quality health care for the poor: Community perceptions on quality care in Uganda. Patient preference and adherence. 2009;3:77 10.2147/PPA.S4091 19936148 PMC2778436

[43] Damme WV, Leemput LV, Por I, Hardeman W, Meessen B. Out‐of‐pocket health expenditure and debt in poor households: evidence from Cambodia. Tropical Medicine & International Health. 2004;9(2):273–80. 10.1046/j.1365-3156.2003.01194.x 15040566

[44] Kwesiga B, Zikusooka CM, Ataguba JE. Assessing catastrophic and impoverishing effects of health care payments in Uganda. BMC health services research. 2015;15(1):30 10.1186/s12913-015-0682-x 25608482 PMC4310024

[45] Chuma J, Maina T. Catastrophic health care spending and impoverishment in Kenya. BMC health services research. 2012;12(1):413 10.1186/1472-6963-12-413 23170770 PMC3561146

[46] Goudge J, Gilson L, Russell S, Gumede T, Mills A. Affordability, availability and acceptability barriers to health care for the chronically ill: longitudinal case studies from South Africa. BMC health services research. 2009;9(1):75 10.1186/1472-6963-9-75 19426533 PMC2694171

[47] Dror DM, Preker AS. Social reinsurance: a new approach to sustainable community health financing: The World Bank; 2002.

[48] Harris B, Goudge J, Ataguba JE, McIntyre D, Nxumalo N, Jikwana S, et al Inequities in access to health care in South Africa. Journal of public health policy. 2011;32 Suppl 1:S102–23. Epub 2011/07/07. 10.1057/jphp.2011.35 . 21730985

[49] Alam K, Mahal A. Economic impacts of health shocks on households in low and middle income countries: a review of the literature. Globalization and health. 2014;10(1):21 10.1186/1744-8603-10-21 24708831 PMC4108100

